# Endomorphin-2- and Neurotensin- Based Chimeric Peptide Attenuates Airway Inflammation in Mouse Model of Nonallergic Asthma

**DOI:** 10.3390/ijms20235935

**Published:** 2019-11-26

**Authors:** Ewelina Russjan, Kryspin Andrzejewski, Dorota Sulejczak, Patrycja Kleczkowska, Katarzyna Kaczyńska

**Affiliations:** 1 Department of Respiration Physiology, Mossakowski Medical Research Centre, Polish Academy of Sciences, 5 Pawińskiego Str., 02-106 Warsaw, Poland; erussjan@imdik.pan.pl (E.R.); kandrzejewski@imdik.pan.pl (K.A.); 2 Department of Experimental Pharmacology, Mossakowski Medical Research Centre, Polish Academy of Sciences, 5 Pawińskiego Str., 02-106 Warsaw, Poland; dsulejczak@imdik.pan.pl; 3 Department of Pharmacodynamics, The Centre for Preclinical Research (CBP), Medical University of Warsaw, 1B Banacha Str., 02-097 Warsaw, Poland; hazufiel@wp.pl

**Keywords:** airway inflammation, non-atopic asthma, hybrid peptide, pro-inflammatory cytokines, sPLA_2_, MDA, NF-κB

## Abstract

We examined anti-inflammatory potency of hybrid peptide-PK20, composed of neurotensin (NT) and endomorphin-2 (EM-2) pharmacophores in a murine model of non-atopic asthma induced by skin sensitization with 2,4-dinitrofluorobenzene and intratracheal challenge of cognate hapten. Mice received intraperitoneally PK20, equimolar mixture of its structural elements (MIX), dexamethasone (DEX), or NaCl. Twenty-four hours following hapten challenge, the measurements of airway responsiveness to methacholine were taken. Bronchoalveolar lavage (BALF) and lungs were collected for further analyses. Treatment with PK20, similarly to dexamethasone, reduced infiltration of inflammatory cells, concentration of mouse mast cell protease, IL-1β, IL-12p40, IL-17A, CXCL1, RANTES in lungs and IL-1α, IL-2, IL-13, and TNF-α in BALF. Simple mixture of NT and EM-2 moieties was less potent. PK20, DEX, and MIX significantly decreased malondialdehyde level and secretory phospholipase 2 activity in lungs. Intensity of NF-κB immunoreactivity was diminished only after PK20 and DEX treatments. Neither PK20 nor mixture of its pharmacophores were as effective as DEX in alleviating airway hyperresponsiveness. PK20 effectively inhibited hapten-induced inflammation and mediator and signaling pathways in a manner seen with dexamethasone. Improved anti-inflammatory potency of the hybrid over the mixture of its moieties shows its preponderance and might pose a promising tool in modulating inflammation in asthma.

## 1. Introduction

Asthma is a chronic disease characterized by airway inflammation, remodeling, and bronchial hyperresponsiveness with usually reversible airflow limitation [1,2]. According to the Global Asthma Report, the prevalence of asthma has increased lately, and over 334 million people worldwide have been reported to be affected [3]. Treatment of both controlled and severe uncontrolled asthma patients poses a substantial economic burden for developed and developing countries [4,5,6]. Inhaled bronchodilators and corticosteroids are currently used to improve asthma condition. However, most of these medications produce unwanted adverse effects, resistance with long-term use, and unresponsiveness [7,8]. The disease is also difficult to treat due to its heterogeneity and different phenotypes involving a multitude of inflammatory mediators [9]. Therefore, there is an urgent need to develop novel anti-inflammatory therapies for the treatment of asthma.

In the last few years, a strong interest has been raised in hybrid compounds encompassing at least two various biologically active fragments, which may simultaneously reach several targets [10,11,12]. One of the hybrid peptides, designated PK20, comprises modified endomorphin-2 (EM-2) and neurotensin (NT) pharmacophores, and it has been shown previously as a highly potent analgesic, as well as a neuroprotective agent [13,14]. Detailed information on the hybrid structure, amino acid modification, and an improved stability can be found elsewhere [13,15]. Both elements of the ligand seem to possess anti-inflammatory potency. NT attenuated oxidative stress and apoptosis in the model of inflammatory bowel disease [16]. The latest study showed the ameliorating action of NT on inflammatory processes and airway hyperreactivity in an experimental asthma model [17]. Likewise, stimulation of opioid receptors resulted in the decrease of edema, plasma extravasation, immune mediators’ level, and tissue damage in various inflammation models [18].

With respect to airways and their innervation, an excitation of opioid receptors by endomorphins has been shown to inhibit cholinergic [19], non-cholinergic, and non-adrenergic bronchoconstriction [20] and the release of pro-inflammatory sensory neuropeptides from capsaicin-sensitive nerves [21] and tachykinergic airway smooth muscle constriction [20]. Therefore, we think that a new therapy with PK20-hybrid comprising EM-2 and NT in the structure offers not only ant-inflammatory potency but also promise of improved bronchodilator activity.

Considering the abovementioned, the aim of the present study was to evaluate the PK20-exerted effects on airway hyperreactivity and inflammation in a mouse model of dinitrofluorobenzene (DNFB) induced non-atopic asthma [22,23]. The effect of the tested hybrid was also compared with glucocorticosteroid dexamethasone and with co-administration of simple mixture of EM-2 and NT (8–13) pharmacophores. The latter was performed to see whether connection of two different elements into one molecule provides further beneficial properties. For these purposes, bronchoalveolar lavage fluid (BALF) and lung tissue were collected for biochemical, immunohistochemical, and histological analyses. 

## 2. Results

### 2.1. Effect of PK20 on DNFB-Induced Inflammatory Cell Infiltration in BALF and Lung Tissue 

**Figure 1:**
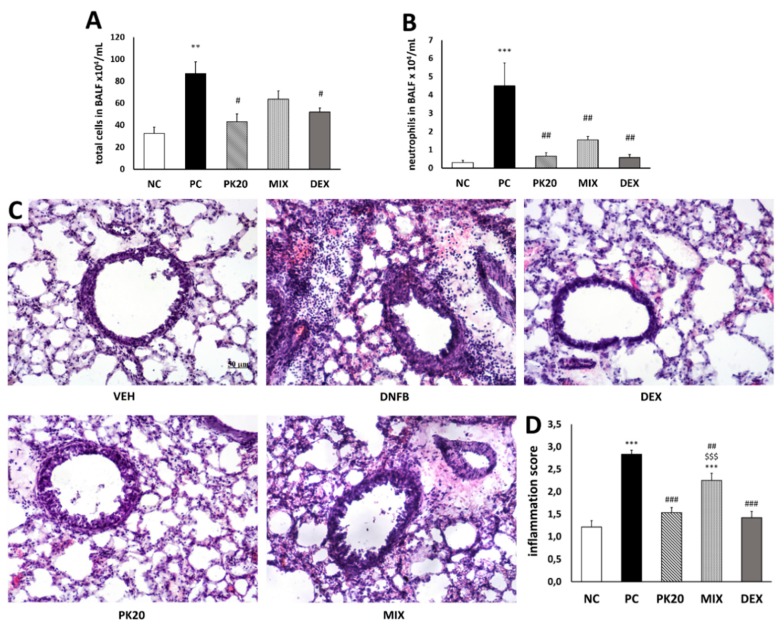
Effect of PK20 on the total number of inflammatory cells (**A**) and neutrophils (**B**) in BALF and effect of PK20 on lung-tissue inflammatory cell infiltration; hematoxylin-and-eosin stained lung sections (**C**) and inflammation score (**D**). Comparison to DNFB-sensitized/DNS-challenged group (positive control; PC) and vehicle-sensitized/DNS-challenged group (negative control; NC) treated with NaCl and to DNFB-sensitized/DNS-challenged groups treated with dexamethasone (DEX) and equimolar mixture of hybrid’s structural elements (MIX). The pictures were made at magnification 10×. All values are the mean ± SEM (*n* = 6–9 in BALF and *n* = 4 in histology study). ** *p* < 0.01, *** *p* < 0.001 vs. NC, # *p* < 0.05, ## *p* < 0.01, ### *p* < 0.001 vs. PC, $$$ *p* < 0.001 vs. PK20 and DEX groups.

Compared with vehicle-sensitized/DNS-challenged group (negative control; NC), DNFB-sensitized/DNS-challenged (positive control; PC) mice showed in BALF a significant increase in the infiltrate of total cells, which consisted of an augmented cell number of macrophages (94%), neutrophils (5%), and lymphocytes (0.8%). The total cell number was significantly lowered only in PK20 and dexamethasone (DEX) groups (Figure 1A). The neutrophil number was significantly reduced in all three groups treated with PK20, mixture of its pharmacophores (MIX), and DEX (Figure 1B). These changes were parallel to histological evaluation, which showed marked peribronchial leukocyte accumulation in the PC group (Figure 1C). The inflammation score was significantly attenuated in all treated groups; however, MIX mice showed significantly higher value in comparison to the PK20 and DEX groups (Figure 1D). 

### 2.2. PK20 Reduces Airway Hyperresponsiveness (AHR)

**Figure 2:**
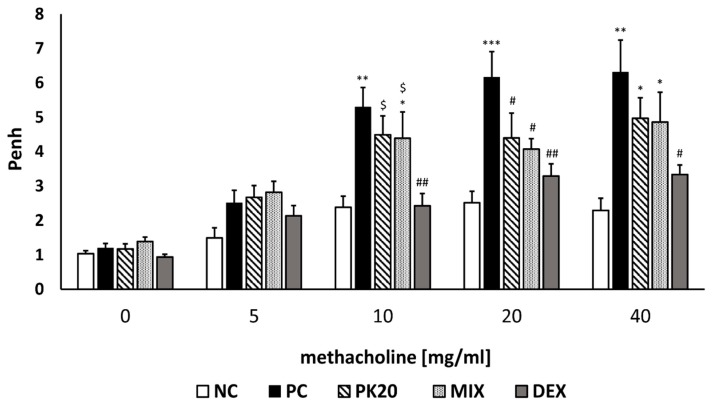
Effect of PK20 on the development of airway hyperreactivity in non-atopic asthma model. Penh responses to increasing concentrations of aerosolized methacholine in DNFB-sensitized/DNS-challenged group (positive control; PC) and vehicle-sensitized/DNS-challenged group (negative control; NC) treated with NaCl and in DNFB-sensitized/DNS-challenged groups treated with PK20, dexamethasone (DEX), and equimolar mixture of hybrid’s structural elements (MIX). All values are the mean ± SEM (*n* = 7–8). * *p* < 0.05, ** *p* < 0.01, *** *p* < 0.001 vs. corresponding NC group. # *p* < 0.05, ## *p* < 0.01, vs. corresponding PC group and $ *p* < 0.05, $$ *p* < 0.01 vs. corresponding DEX group.

To assess whether PK20 had a beneficial effect on AHR during an inflammatory response in lungs in DNFB-induced asthma, we exposed mice to the increasing doses of methacholine (MCh) aerosol in whole-body plethysmograph. Penh (enhanced pause) was measured in this noninvasive method at 24 h post-challenge. DNFB-sensitized/DNS-challenged mice displayed increased Penh to growing doses of MCh as compared to mice in the vehicle-sensitized/DNS-challenged group (Figure 2). Treatment with the hybrid peptide PK20 and the mixture of its pharmacophores significantly reduced AHR in DNFB-sensitized/DNS-challenged mice at 20 mg/mL of inhaled MCh. DEX-treated mice exhibited lowered Penh at all higher doses of MCh in comparison to the PC group (Figure 2).

### 2.3. Effect of PK20 on DNFB-Induced Pro-Inflammatory Cytokine and Chemokine Production

**Figure 3:**
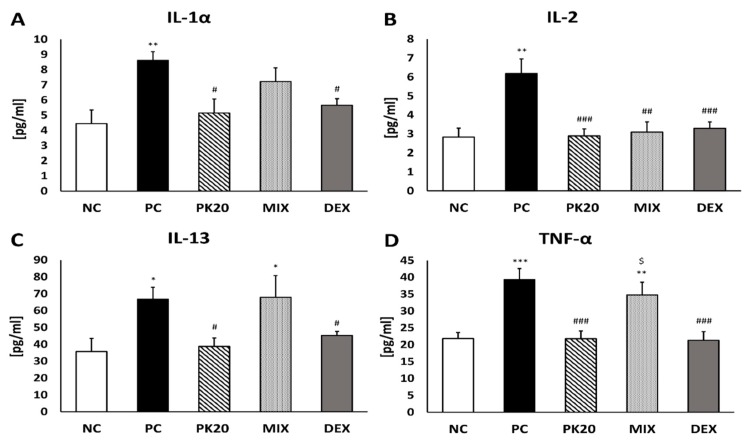
Concentration of pro-inflammatory cytokines in BALF: IL-1α (**A**), IL-2 (**B**), IL-13 (**C**), and TNF-α (**D**) in DNFB-sensitized/DNS-challenged mice after treatment with PK20, mixture of its structural elements (MIX), and dexamethasone (DEX). Comparison to DNFB-sensitized/DNS-challenged (positive control; PC) and vehicle-sensitized/DNS-challenged group (negative control; NC) treated with NaCl. All values are the mean ± SEM (*n* = 5–9). * *p* < 0.05, ** *p* < 0.01, *** *p* < 0.001 compared with NC group, # *p* < 0.05, ## *p* < 0.01, ### *p* < 0.001 compared with the PC group, $ *p* < 0.001 vs. PK20 and DEX groups.

Measurement of pro-inflammatory cytokine content was performed 24 h after intratracheal DNS challenge in BALF and lung homogenates of DNFB or vehicle-sensitized mice, to determine whether treatment with PK20 is able to influence their production. The levels of IL-1α, IL-2, IL-13, and TNF-α were significantly increased in BALF of DNFB-sensitized/DNS-challenged mice (PC) compared to NC group (Figure 3). The levels of all cytokines were significantly decreased after PK20 and DEX treatment, whereas the co-administration of PK20′s opioid- and NT-like pharmacophores resulted in decreased content of IL-2, solely (Figure 3B).

**Figure 4:**
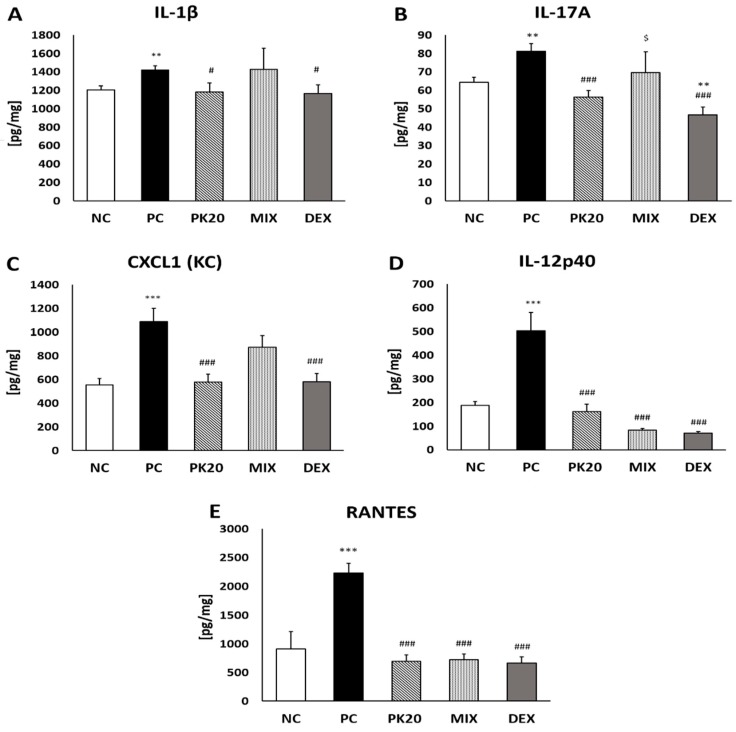
Concentration of pro-inflammatory cytokines in lung-tissue homogenates: IL-1β (**A**), IL-17A (**B**), IL-12p40 (**C**), KC (**D**), and RANTES (**E**) in DNFB-sensitized/DNS-challenged mice after treatment with PK20, mixture of its structural elements (MIX), and dexamethasone (DEX). Comparison to DNFB-sensitized/DNS-challenged (positive control; PC) and vehicle-sensitized/DNS-challenged group (negative control; NC) treated with NaCl. All values are the mean ± SEM (*n* = 5–7). ** *p* < 0.01, *** *p* < 0.001 compared with NC group. # *p* < 0.05, ### *p* < 0.001 compared with the PC group; $ *p* < 0.05 vs. DEX.

In lung homogenates PK20 and DEX in similar degree reduced levels of IL-1β, IL-17A, IL-12p40, CXCL1 (KC), and RANTES in comparison to the PC group (Figure 4). Treatment with the mixture of PK20 pharmacophores was effective only in decreasing content of IL-12p40 and RANTES (Figure 4D,E).

### 2.4. Effect of PK20 Treatment on Mouse Mast Cell Protease (MCPT 1) Level in Lungs

**Figure 5:**
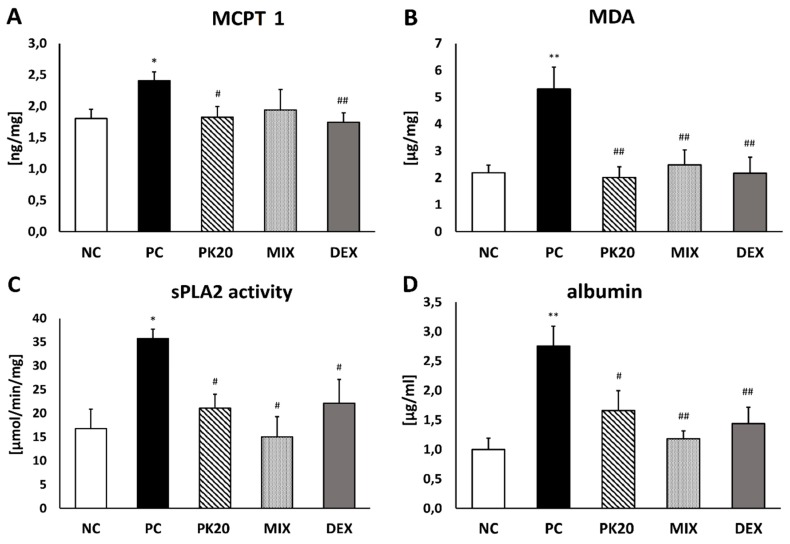
Concentration of (**A**) mouse mast cell protease-1 (MCPT 1), (**B**) malondialdehyde (MDA), and (**C**) activity of secreted phospholipase A_2_ (sPLA2) in lung-tissue homogenates and (**D**) level of albumins in BALF of DNFB-sensitized/DNS-challenged mice after treatment with PK20, mixture of its structural elements (MIX), and dexamethasone (DEX). Comparison to DNFB-sensitized/DNS-challenged (positive control; PC) and vehicle-sensitized/DNS-challenged group (negative control; NC) treated with NaCl. All values are the mean ± SEM (*n* = 5–8). * *p* < 0.05, ** *p* < 0.01 compared with NC group. # *p* < 0.05, ## *p* < 0.01 compared with the PC group.

DNFB-sensitized/DNS-challenged mice exhibited a significantly higher level of MCPT 1 in lung tissue compared with vehicle-sensitized/DNS-challenged ones. Only PK20 and DEX treatments were effective in lowering the content of mast cell MCPT 1 (Figure 5A).

### 2.5. Effect of PK20 Treatment on Malondialdehyde Level (MDA) in Lungs

Sensitization and challenge with DNFB/DNS showed a statistically significant enhancement in MDA production compared with NC mice (Figure 5B). The content of an oxidative stress marker was significantly reduced in all treated groups (PK20, MIX, and DEX) and similar to the level present in NC mice (Figure 5B).

### 2.6. Effect of PK20 Treatment on Activity of Secreted Phospholipase A_2_ (sPLA2) in Lungs

sPLA2 activity was significantly reduced in lungs of PK20-, MIX-, and DEX-treated groups compared to PC mice. The levels were comparable to the sPLA2 activity in vehicle-sensitized/DNS-challenged negative controls (Figure 5C).

### 2.7. Effect of PK20 Treatment on Albumin Concentration in BALF

Albumin concentration expressed in mg of total protein assessing pulmonary vascular leakage was significantly enhanced in PC mice compared to negative controls. The values were significantly lowered in PK20-, MIX-, and DEX-injected animals (Figure 5D). 

### 2.8. Effect of PK20 on Lung Nuclear Factor Kappa B (NF-κB) Immunohistochemistry

**Figure 6:**
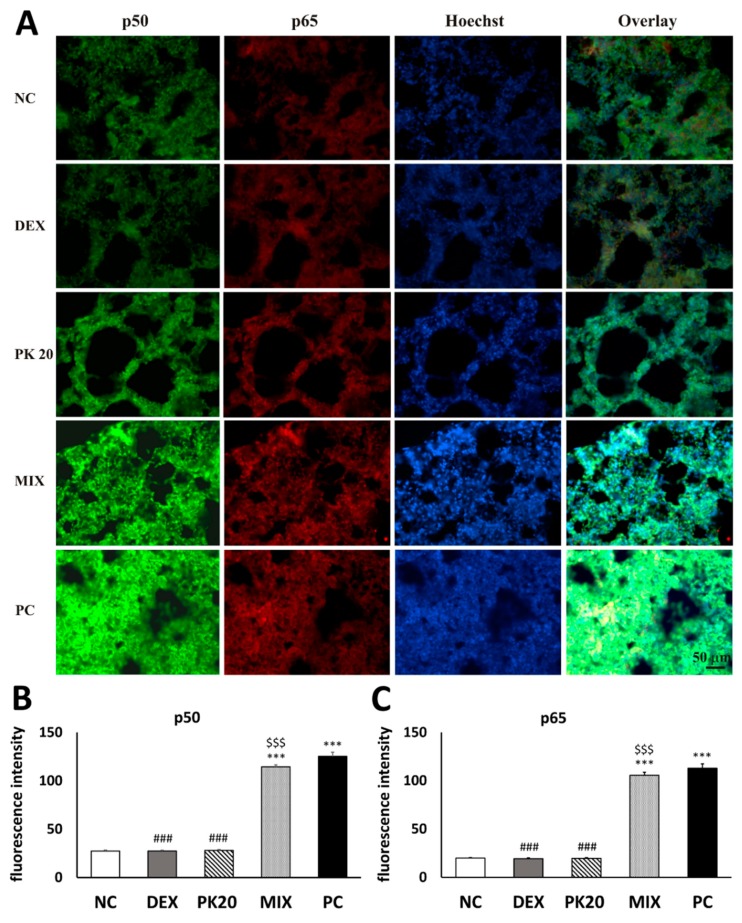
Effect of PK20 on NF-κB expression in DNFB-induced asthma in mice. Immunofluorescent double labeling of NF-κB p50 (green) and NF-κB p65 (red) subunits and Hoechst stain of the nuclei in lung sections from each treatment group was performed (**A**). The intensity of immunostaining was measured in grey scale and presented on panel (**B**) (p50) and (**C**) (p65). The treatment groups are: vehicle-sensitized/DNS-challenged group (negative control; NC) treated with NaCl; DNFB-sensitized/DNS-challenged group treated with NaCl (positive control; PC); and DNFB-sensitized/DNS-challenged groups treated with PK20, dexamethasone (DEX) and equimolar mixture of hybrid’s structural elements (MIX). All values are mean ± SEM (*n* = 4). *** *p* < 0.001 vs. corresponding NC group, ### *p* < 0.001 vs. corresponding PC group and $$$ *p* < 0.001 vs. corresponding PK20 and DEX groups.

Challenge of DNS into airways of DNFB-sensitized mice significantly increased immunofluorescence signal for NF-κB in comparison to the vehicle-sensitized NC group (Figure 6A). In contrary to MIX, PK20 and DEX treatment apparently blunted the signal, which indicates that the NF-κB signaling pathway may be involved in the anti-inflammatory activity of both chemicals (Figure 6A–C). Note the massive enlargement of the pulmonary interalveolar septa in the mice from MIX and PC groups and more intensive lung NF-κB p50 and p65 staining in comparison to negative control, PK20-, and DEX-treated animals. This significantly increased NF-κB p50 and p65 expression most probably resulted in augmented inflammatory response present in PC and MIX animals.

## 3. Discussion

In the murine model of non-atopic asthma, we demonstrated that the hybrid peptide engineered from pharmacophores of NT- and EM-2-based analogs may serve as a potent anti-inflammatory agent affecting a number of inflammation markers, and its potency is comparable to the effects mediated by the dexamethasone. However, glucocorticosteroid showed a stronger impact on attenuation of airway hyperreactivity. Through the comparison of the effects of chimera with simple equimolar mixture of its structural elements, we also confirmed the hypothesis that two drugs combined into one entity may be characterized by improved therapeutic activity. Indeed, PK20 treatment occurred to be more effective in substantial reduction of inflammatory cell influx, including neutrophils, typical cells for nonallergic asthma [22,24]. Reduced inflammatory cell influx was associated with diminished production of pro-inflammatory cytokines and chemokines in BALF and lung tissue. These effects were comparable to DEX, and better than MIX treatment, in which a decrease of only IL-2, IL-12p40, and RANTES was noted.

Reduction of IL-1β, TNF-α, IL-17A, and CXCL1 (KC) was parallel to PK20 evoked attenuation of macrophage and neutrophil infiltrate. Cytokines, such as TNF-α and IL-1β, augmented in sputum and BALF of asthmatics, are well-known for their role in amplifying inflammation [25] and promoting neutrophil chemotaxis [26,27,28]. Potency of IL-1β in eliciting neutrophil accumulation in rat BALF was shown after intratracheal interleukin administration, and this was concomitant with an increased airway responsiveness to bradykinin stimuli [29]. Importance of both IL-1 cytokines, IL-1β and IL-1α, in AHR and recruitment of inflammatory cells to the lung was evidenced after blockade with neutralizing antibodies, resulting in attenuated phenotype of murine model of asthma [30].

Contribution of TNF-α to airway hyperreactivity and airway inflammation mediated by neutrophils was demonstrated previously in normal subjects and ovalbumin and DNFB-sensitized mice [22,31,32]. Elevated levels of IL-17A displayed in sputum of asthmatics were correlated not only with neutrophilia, but also with an airway hyperresponsiveness provoked with methacholine [33]. Further reports supported the role of IL-17A in a neutrophil- and macrophage-mediated inflammation [34,35], appearing also in our asthma model. Likewise, chemokines CXCL1 and RANTES, decreased due to the hybrid administration, have been described to be potent neutrophil chemoattractant [36,37,38]. Cytokines such as IL-2, IL-12p40, and IL-13 increased in the present study and reduced by the hybrid are documented to be important mediators of asthma. IL-2 was elevated in BALF of nonallergic asthma patients [39]; moreover, expression of IL-2 mRNA was enhanced in infiltrated BALF cells in steroid-resistant asthmatics [40]. IL-12p40 overexpression in airway epithelial cells in asthma patients was linked with airway macrophages accumulation [41]. IL-13 is a pro-inflammatory interleukin in asthma [42], which, similarly to our study, was increased in BALF in the allergic model and reduced after treatment with dexamethasone [43]. IL-13 is thought to be the type 2 cytokine and key mediator of allergic asthma, yet it is considered to act via the pathway independent of immunoglobulin E and eosinophils [44]. Elevated mRNA encoding IL-13 has been reported in the bronchial mucosa of atopic and non-atopic asthmatics [45].

Oxidative stress and overproduction of reactive oxygen species (ROS) associated with augmented inflammatory response has been displayed in human asthma and experimental models [46]. In the present study, we showed that DNFB sensitization enhanced the level of MDA production, which was remarkably reduced by the treatment with PK20, DEX, and a mixture of pharmacophores. This was linked with a decreased number of recruited cells in BALF, which in turn are responsible for ROS production [47,48] and with the lessened levels of IL-1, RANTES and TNF-α, which are reported to enhance ROS generation [49,50,51]. Furthermore, augmented oxidative stress and increased release of pro-inflammatory cytokines are known to activate secreted phospholipase A_2_ (sPLA_2_), enzyme-regulating eicosanoid synthesis, airway inflammation, and AHR in asthma [52]. Secreted PLA2 group X was demonstrated to be overexpressed in airway epithelial cells and macrophages of asthmatics [53,54]. Further, it was evidenced that sPLA_2_ increase can be provoked with allergen inhalation in humans and mice [55]. In fact, sPLA_2_ activity was increased in our positive control group and reduced by pretreatment with all tested compounds. TNF-α, IL-1β, IL-17A, and IL-13 are cytokines, which decrease in PK20 and DEX groups is correlated with sPLA_2_ activity reduction. All of them are implicated in asthma and has been demonstrated to upregulate sPLA_2_ [54]. 

Oxidative stress is known to activate NF-κB, which is a pleiotropic transcription factor considered to be a critical signal in evoking an inflammatory response in the lung during the pathogenesis of asthma [56,57]. Pro-inflammatory stimuli enhance expression of genes encoding inflammatory mediators, such as cytokines and chemokines, important in the recruitment of inflammatory cells. Consistently in the current study, NF-κB immunoreactivity was increased in the DNFB-sensitized/DNS-challenged group, yet in DEX- and PK20- treated mice, it was significantly reduced in contrary to the MIX group. Dexamethasone’s potency in inhibiting NF-κB immunoreactivity was also reported previously [43]. PK20-induced suppression of NF-κB was consistent with reduced oxidative stress and decreased expression of NF-κB-mediated pro-inflammatory cytokines and chemokines, which contribute to attenuated recruitment of inflammatory cells. All of these findings suggest that the NF-κB signaling pathway might be involved in the alleviating effect of the hybrid on hapten-induced asthma. The limitation of our study is that we measured NF-κB expression by only using immunohistochemistry; therefore, further experiments investigating NF-κB activation, that is, the levels of phosphorylated units and nuclear translocation, via Western blot are needed. 

An important possibility of testing our hybrid compound and its effect on the signaling pathways involved in airway inflammation and hyperresponsiveness, such as TNF-α/IL-13, NF-κB, MAPK, c-fos, and c-jun seems to be in vitro airway organ culture targeted in the earlier studies [58,59], which can be considered in our future research.

A consistent finding of our study was improved lung vascular integrity produced with all compared substances, evidenced by decreased albumins in BALF. This might be associated with decreased migration of leukocytes, such as neutrophils and monocytes, to the lungs, evidenced in our study and reported previously to contribute to increased lung permeability [60,61]. Another marker of inflammation attenuated by PK20 and DEX in the present study was mouse mast cell protease (MCPT 1), biomarker of mast cell degranulation. The key role of mast cells in the development of non-atopic asthma induced with DNFB has been previously evidenced [62]. Their contribution to neutrophil recruitment through TNF-α was also displayed in delayed-type hypersensitivity reaction evoked with cognate hapten by Biedermann et al. [63].

In the current study, we also examined airway responsiveness to methacholine inhalation, measured by whole-body plethysmography. Validity of Penh is still under debate, as it is considered to reflect pattern of breathing rather than lung mechanics [64]. However, data published by different groups have displayed similar changes in Penh and lung resistance measured invasively [64,65,66,67]. Although Penh is affected by airway resistance only to some extent [68], we do think that this noninvasive measurement has some value in depicting airway responsiveness, at least for screening purposes. More important, substantial increases in Penh in reaction to methacholine appeared only in DNFB-sensitized/DNS-challenged mice in contrast to vehicle-sensitized/DNS-challenged ones. Dexamethasone treatment effectively reduced Penh to all doses of methacholine in comparison to positive controls. The chimeric peptide, as well as the mixture of its structural elements, achieved significant reduction solely at a dose of 20 mg/mL, and it was not as effective as DEX. One would expect that similar reduction of several markers of inflammation described above, such as BALF cell influx, cytokine, MDA, and sPLA_2_ activity, should translate into decreased AHR. However, it is known that there is no consistent relationship between airway hyperresponsiveness to bronchoconstrictor agents and inflammation [69,70]. Furthermore, Tränkner et al. [71] showed that hyperreactive bronchoconstriction can be completely dissociated from the inflammatory immune response and ablated after silencing of the vagal sensory neurons, expressing transient receptor potential vanilloid 1 (TRPV1) ion channel. It seems that PK20’s being potent in suppressing inflammation is able only partially to control airway hyperresponsiveness. 

It is assumed that hybrid can exert its anti-inflammatory action via opioid receptors expressed by cells associated with inflammation, such as macrophages, mast cells, and fibroblasts [72]. Endomorphins and opioid receptors have been localized within airway smooth muscles, nerves, epithelium, and mucus glands [20]. In the respiratory system, they have been described as potent inhibitors of tachykinergic and cholinergic constriction, neurogenic mucus secretion, and goblet cells secretion [20,73]. In the review by Stein and Kuchler [18], reduction of edema, plasma extravasation, cytokine release, and tissue damage evoked by opioid-receptor activation were described in various models of inflammation. When it comes to neurotensin, its anti-inflammatory activity is far from clear. In fact, based on several studies, this neuropeptide is indicated to stimulate mast cell degranulation [74], neutrophil adherence to bronchial epithelial cells [75], and genetically modified mice deprived of production of NT had attenuated inflammatory response [76]. On the other hand, NT-treated animals in the model of inflammatory bowel disease showed a protective effect on the intestines related to anti-inflammatory, antioxidant, and anti-apoptotic actions [16]. The latest study showed that NT is a potent downregulator of inflammatory and hyperreactivity response in the nonallergic asthma model [17]. We should keep in mind that the activity exerted by the chimeric peptide PK20, used in our study, cannot be considered as a simple conjunction of activities appropriate to its parent peptides or, more precisely, pharmacophores. Hybridization of two modified structures either of EM-2 or NT (8–13) into one molecule may bring new and diverging physical and chemical properties. In the present study, we showed beneficial effect of PK20 on many markers of inflammation characteristic for asthma, such as attenuated production of MDA and cytokines, reduced activity of sPLA_2_ and expression of NF-κB, and diminished level of MCPT 1. We also made a comparison between beneficial effects of PK20 and a mixture of its moieties, as still multicomponent drugs and/or polypharmacy play a leading role in patients’ treatment, though it is associated with adverse outcomes and drug reactions. Our experiments revealed that PK20 predominates in reduction of majority of examined markers of inflammation, which may be explained by its improved stability, bioavailability, and possibly new properties, which resulted in better anti-inflammatory activity. 

Overall, the present study demonstrated that chimeric peptide-PK20 attenuated DNFB induced upregulation of asthma symptoms possibly via suppression of sPLA_2_ and NF-κB pathways. Improved anti-inflammatory potency of the hybrid shows the advantage of application of innovative chimeric compounds over the simple mixture of its moieties.

## 4. Materials and Methods 

### 4.1. Drugs and Reagents

PK20 (H–Dmt–D–Lys–Phe–Phe–Lys–Lys–Pro–Phe–Tle–Leu–OH) was synthesized by using solid-phase peptide synthesis according to the method of Merrifield with Fmoc approach [77] in the Department of Neuropeptides. Chemicals and reagents were purchased from commercial companies: 1-fluoro-2, 4-dinitrobenzene (DNFB, No. 556971), dexamethasone (No. D4902), dinitrobenzene sulfonic acid (DNS, No. D1529), protease inhibitor cocktail (No. S8830), and TX-100 (No. X100) were obtained from Sigma-Aldrich (Poznań, Poland). Ketamine, xylazine, and pentobarbital sodium were purchased from Biowet (Puławy, Poland).

### 4.2. Animals

Animals were obtained from the Animal House of Mossakowski Medical Research Centre, Polish Academy of Sciences. Male Balb/c mice of 7–8 weeks of age were used. Animal procedures were performed in accordance with institutional guidelines accepted by the IV Warsaw Ethics Commission for the Care and Use of Laboratory Animals for Experimental Procedures (permit number: 57/2014, date of approval: 16 January 2015). Procedures were conformed to guidelines published in the European directive 2010/63/EU on the protection of animals used for scientific purposes.

### 4.3. DNFB-Induced Experimental Asthma

On day 1, mice were epicutaneously sensitized with either 0.5% DNFB dissolved in acetone and olive oil (4:1) or vehicle control onto shaved abdominal skin (50 μL) and paws (50 μL divided on four paws). On the next day, DNFB/vehicle was applied to the thorax alone. On day 6, mice were challenged intratracheally with 50 μL of DNS (0.6%), water soluble hapten of DNFB. The sensitization and hapten challenge were performed under intramuscular anesthesia: ketamine (70 mg/kg) and xylazine (10mg/kg). Twenty-four hours from the challenge airway hyperresponsiveness to nebulized methacholine was measured. Afterward, the mice were sacrificed with an overdose of pentobarbital sodium (150–200 mg/kg IP) and bronchoalveolar lavage fluid (BALF), and lungs were collected for histology and further analyses.

### 4.4. Experimental Groups

The experimental mice were randomly assigned into following groups: —treated with 0.9% NaCl, vehicle-sensitized/DNS-challenged, negative control (NC); —treated with NaCl, DNFB-sensitized/DNS-challenged, positive control (PC); —PK20 treated, DNFB-sensitized/DNS-challenged (PK20); —treated with mixture of PK20 structural pharmacophores, DNFB-sensitized/DNS-challenged (MIX); —dexamethasone treated, DNFB-sensitized/DNS-challenged (DEX). The dose of PK20 (10 mg/kg i.p.) was selected on the basis of an analgesic study performed by Kleczkowska et al. [13] and our neurotensin-based study [17]. PK20 applied via IP route of injection was previously shown to be safe, without visible adverse side effects [78]. PK20 prepared freshly from powder, (dissolved in NaCl) was administered twice (2 and 8 h after DNS challenge) in volumes of 100 μL. All groups received equivalent volumes and route of injection: NaCl—control groups (NC, PC), equimolar amount of mixture of NT and EM-2 pharmacophores—MIX group, and 4 mg/kg of dexamethasone received DEX group (all chemicals were dissolved in NaCl).

### 4.5. Measurement of Airway Hyperresponsiveness

Airway hyperresponsiveness to nebulized methacholine (MCh, No. A2251) was measured 24 h after DNS challenge by a whole-body barometric plethysmography (Buxco Electronics, Inc., Wilmington, NC, USA). Mice were put in the plethysmograph chamber for 10 min to adaptation. After stabilization, they were exposed to aerosolized saline as a control and every 20 min to increasing doses of nebulized methacholine for 30 s each (5, 10, 20, and 40 mg/mL). The enhanced pause (Penh) was recorded and averaged over 3 min, following each nebulization, and used as an index of airway reactivity. 

### 4.6. Total and Differential Cell Counts in Bronchoalveolar Lavage Fluid

After sacrificing the animals, the trachea of each mouse was cannulated. Bronchoalveolar lavages were carried out by injecting and withdrawing via a cannula 4 × 1 mL of phosphate buffered saline (PBS). The first 1 mL contained protease inhibitor cocktail. Collected lavages were centrifuged (1500 rpm, 10 min) to isolate BALF cells and to remove supernatant. Supernatant from the first 1 mL was frozen and used for further analysis. Sediments of the fourth lavages were pooled and re-suspended by 150 μL of PBS. Total cells were counted by using a Bürker-Türk chamber. Remaining cell suspensions were cytocentrifuged (Thermo Shandon, Cambridge, UK) at 700 rpm for 10 min onto microscopic slides. Air-dried preparations were fixed and stained with a fast-staining kit based on eosin and azure solutions (No. AB123H, Hemastain, Analab, Warsaw, Poland), in order to differentiate morphologically the leukocyte population. Differential cell count on at least 500 cells from each sample was performed under light microscope. Results are expressed as the number of mononuclear cells (macrophages and leukocytes) and neutrophils per 1 mL of BALF.

### 4.7. Cytokine and Chemokine Quantification

Immediately after BALF collection, the lungs were removed and cleaned. Lungs and first 1 mL of BALF containing protease inhibitor cocktail were frozen in liquid nitrogen and stored at −80 °C. For further analysis, lungs were homogenized under liquid nitrogen and taken up in 500 μL of PBS with protease inhibitor cocktail and 1% of TX-100. Homogenates were centrifuged (14,000 rpm, 10 min, 4 °C), and the supernatants were used for cytokines measurement. Measurement of IL-1α, IL-2, IL-13, TNF-α, IL-1β, IL-12p40, IL-17A, CXCL1 (KC), and RANTES in BALF and lung homogenates was performed by using a Bio-Plex pro mouse cytokine panel for inflammatory cytokines (No. M60009 RDPD, Bio-Rad, Warsaw, Poland) according to the manufacturer’s protocol. Beads were analyzed at a Bio-Plex 200 Platform (Luminex, Bio-Rad, Warsaw, Poland).

### 4.8. Measurement of Oxidative Stress (Malondialdehyde Level) in Lung Tissue

Malondialdehyde (MDA), aldehydic secondary product of lipid peroxidation, is a marker of oxidative stress. The determination of MDA concentration was performed in supernatants from lung tissue, using a commercially available kit (No. STA-832, OxiSelect™ MDA, Cell Biolabs, Inc., San Diego, CA, USA).

### 4.9. Measurement of Mouse Mast Cell Protease Level (MCPT 1) in Lung Tissue

A commercially available enzyme-linked immunosorbent assay—the Mouse MCP-1 ELISA Ready-SET kit (No. 88-7503, eBioscience, San Diego, CA, USA) was used for the quantification of MCPT 1 in lung-tissue supernatants. 

### 4.10. Measurement of Secretory Phospholipase 2 (PLA2) Activity

The sPLA2 activity was measured in the lung supernatant by using a sPLA2 kit (No. 765001, Caymann Chemicals, Ann Arbor, MI, USA) according to the manufacturer’s instructions and expressed in μmol/min/mg. The enzyme activity was read for 10 min at 414 nm, using an ELISA plate reader.

### 4.11. Determination of Total Protein and Albumin Concentration

Total protein assay was performed in lung supernatants and BALF with protein assay kit (No. 500-0006, Bio-Rad, Warsaw, Poland). Albumin levels were measured in collected BALF with a murine-specific albumin ELISA kit (No. E99-134, Bethyl Laboratories, Montgomery, TX, USA). All the measurements were performed according to the manufacturer’s instructions. The sPLA2 activity and the content of cytokines, MDA, and MCPT 1 in lungs and albumins in BALF were expressed in units of weight per mg of total protein.

### 4.12. Lung Histology

The lungs were fixed for 7 days in the 4% paraformaldehyde in 0.1 M phosphate buffer at 4 °C. Afterward, the tissue was immersed in 20% (*w/v*) sucrose solutions in PBS and cut with a cryostat (CM 1850 UV, Leica, Germany) on 20 μm thick glass-mounted sections. After staining with eosin (No. 1.09844) and hematoxylin (No. 1.50174, Merck Millipore, Warsaw, Poland), they were examined under a light microscope (Olympus, Japan). To assess the severity of the immune cells’ infiltration, peribronchial and perivascular cell counts were performed based on the 4-point score system described in detail by Tournoy et al. [79], where higher scores designated more severe inflammation (more inflammatory cell rings around bronchi or vessels). 

### 4.13. NF-κB Immunohistochemistry

The studied lung sections, prepared as described in Section 4.12, were subjected to double labeling for NF-κB p50 and NF-κB p65 peptides. After preincubation with 3% normal goat serum solution in PBS with 0.2% Triton X-100 (PBST), the samples were subsequently incubated for 1 h at 37 °C with PBST containing 1% normal goat serum and primary antibodies (Abs): murine monoclonal Ab against NF-κB p65 (No. sc-8008, Santa Cruz Biotechnology, Dallas, TX, USA, dil. 1:300) and rabbit polyclonal Ab against NF-κB p50 (No. sc-114, Santa Cruz Biotechnology, Dallas, TX, USA, dil. 1:3400). Next, the sections were washed three times in PBS and incubated for 1 h at 37 °C, with a mixture of the fluorescent dye-conjugated secondary antibodies: goat anti-mouse Alexa Fluor 594 (No. A11020, Invitrogen-Molecular Probes; dil. 1:100) and goat anti-rabbit Alexa Fluor 488 (No. A11070, Invitrogen-Molecular Probes; dil. 1:100). In the next stage, the samples were washed three times with PBS and dried.

Immunolabelled sections were washed in PBS and incubated in 1 µg/mL of PBS solution of Hoechst stain (bisbenzimide dye, No. 33258, Sigma-Aldrich, Poznań, Poland) for 2 min, at room temperature. The stain was then drained off and cover-slipped with Vectashield Mounting Medium for fluorescence microscopy (No. H1000, Vector Laboratories, Inc., Burlingame, CA, USA). Finally, the sections were analyzed with a fluorescent microscope (Nikon, Japan) and photographed with a CCD camera (Nikon, Japan). The specialist who assessed the material was blinded to the identity of the samples. Specificity of the immunolabelling was verified by performing a negative control staining procedure, in which the primary antibodies were omitted in the incubation mixture. The intensity of p65, as well as p50 immunostaining, was measured as an optical density (OD) level in grayscale values (8-bit scale), using Scion computer software (NIH, Frederick, MD, USA). Analysis was carried on within the lung sections framed by microscopic observation field. Three frames of alveolar septum per section and three sections per each mouse from all investigated groups were analyzed. Background staining was sampled on each frame individually and was detracted from the values of the cellular staining. For statistical analysis, the average gray-level value from all the measured sections of each mouse from all investigated groups was used. 

### 4.14. Statistical Analysis

Data were presented as means ± standard error of mean (SEM). Differences between the mean values of the normally distributed data were assessed by using one-way analysis of variance (ANOVA; Newman–Keuls post hoc test) and Student’s *t*-test. The *p*-values less than 0.05 were considered to be statistically significant. All data manipulations and statistical analysis were conducted by the usage of STATISTICA 12 (StatSoft, Kraków, Poland).
